# Long-term exposure to “low-dose” bisphenol A decreases mitochondrial DNA copy number, and accelerates telomere shortening in human CD8 + T cells

**DOI:** 10.1038/s41598-020-72546-x

**Published:** 2020-09-25

**Authors:** Hoai Thi Thu Tran, Corinna Herz, Evelyn Lamy

**Affiliations:** 1 Molecular Preventive Medicine, University Medical Center and Faculty of Medicine, University of Freiburg, 79106 Freiburg, Germany; 2 Pharmaceutical Bioinformatics, Institute of Pharmaceutical Sciences, Faculty of Chemistry and Pharmacy, Albert-Ludwigs-University, Freiburg, Germany

**Keywords:** Cancer, Immunological disorders, Immunology, Environmental sciences, Diseases, Endocrinology

## Abstract

Exposure to the endocrine disruptor bisphenol A (BPA) has been linked with immune disorders and increased tumour risk. Our previous work in activated human peripheral blood mononuclear cells demonstrated that exposure to “low-dose” BPA diminished telomerase activity via an ER/GPR30-ERK signalling pathway. Leukocyte telomerase activity and telomere maintenance are crucial for normal immune function and homeostasis. We thus here further studied the effects of BPA on human T cell subpopulations. Exposure to 0.3–3 nM BPA, i. e. at doses in the realm of human exposure, notably reduced telomerase activity in activated CD8 + T but not CD4 + T cells in a non-monotonic response pattern as determined by the TRAP-ELISA assay. Under long-term BPA exposure, significant telomere length shortening, reduction in mitochondrial DNA copy number, cell proliferation and IFN-γ as well as hTERT protein suppression could be observed in CD8 + lymphocytes, as analysed by qRT-PCR, flow cytometry and western blot analysis. This study extends our previous in vitro findings that “low-dose” BPA has potential negative effects on healthy human cytotoxic T cell response. These results might merit some special attention to further investigate chronic BPA exposure in the context of adaptive immune response dysfunction and early onset of cancer in man.

## Introduction

Bisphenol A (BPA) is a plasticizer used for the production of food contact applications in such a wide range that daily “low-dose” exposure to this compound is inevitable. Besides oral intake, BPA is absorbed through the skin, mainly from receipts and personal care products and inhaled as a contaminant in dust^1^.

BPA has been found in the majority of the human population with unconjugated circulating levels in the range of 0.1- 2 ng/ml (0.4–8.8 nM)^2^. To date there are thousands of scientific studies of BPA, but whether this industrial chemical poses a human health hazard remains “controversial”. Both the European Food Safety Authority (EFSA) and the European Chemical Agency (ECHA) agree that BPA has endocrine disrupting properties^3,4^. BPA acts as an agonist at estrogen receptors (ERs) and mediates responses similar to or even stronger than estradiol (E2)^5^. It also targets to androgen, thyroid hormone and G-protein coupled receptors (GPCRs) at a broad concentration range and non-monotonic cell responses have been observed following increasing BPA levels^6–8^. Traditional toxicology test results provide little or no evidence of harm, and at current exposure levels BPA is assumed to pose only a low health concern for consumers from all sources^1^. Results from independent investigators have reported “low-dose” adverse effects, i. e. at doses in the realm of human exposure, though^9,10^. To date, a remarkable number of in vitro and in vivo studies on “low-dose” BPA exposure have identified a potential risk of BPA for adverse health outcomes^11,12^. Using animals and humanized animal models, Prins et al.^13–15^ proposed that “low-dose” BPA exposure may enhance the cancer risk with ageing, and reduce tumour sensitivity to treatment which is in line with reported cell-based studies^16,17^. Seachrist et al*.*^18^ provided a critical discussion on whether early-life BPA exposure could increase the susceptibility to breast or prostate cancer or haematopoietic malignancies. BPA has been shown to modulate the immune function either via stimulating or suppressing the immune response to antigens and thus potentially promote immune-related diseases including cancer^19–21^. Ménard et al.^22,23^ also reported that BPA might affect the immune system in animals.

Peripheral blood telomerase activity and telomere maintenance are crucial for immune function and homeostasis. Low telomerase activity and shortened telomeres have been reported with advanced aging but also under conditions of chronic stress^24^, chronic inflammation^25^ and environmental exposure to chemicals^26^. Altered telomerase enzyme activity as well as short telomeres have been proposed as risk factors for immune system dysfunction^27,28^. This in turn may ultimately contribute to initiation of malignant tumour formation^29^.

Our previous study reported on the potential negative impact of “low-dose” BPA exposure on human PBMC via persistent down-regulation of telomerase activity^7^. Inhibition of cell proliferation and significant DNA damage were evident after 21 days of continuous “low-dose” BPA exposure in this study^7^. Additionally, BPA altered the cytokine secretion of PBMC upon mitogen stimulation^7^. In the present study, we aimed to further characterize the impact of “low-dose” BPA exposure observed in PBMC by using freshly isolated T cell subpopulations of healthy donors for short and long-term BPA treatment. We focused then on cell function and parameters associated with ageing. In accordance with the previous findings, BPA suppressed telomerase activity in CD8 + T cell subsets and impaired DNA repair capacity upon short-term exposure. Besides telomerase suppression, long-term treatment significantly diminished cell proliferation, telomere length, mitochondrial DNA (mtDNA) copy number and anti-viral IFN-γ production.

## Results

### Short-term “low-dose” BPA exposure inhibited telomerase activity and impaired cell repair capacity in CD8 + T-cells

**Figure 1:**
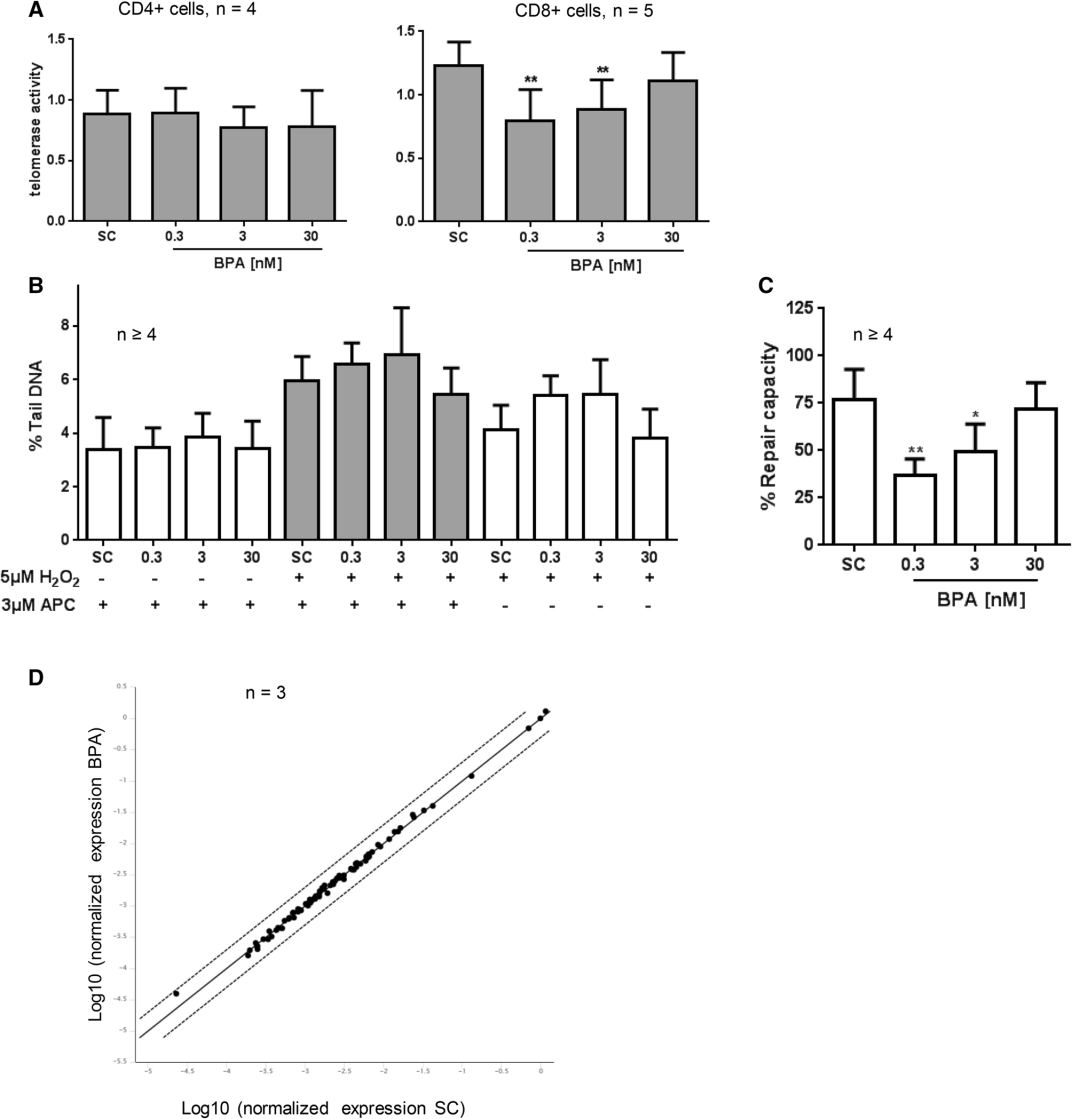
Short-term effect of “low dose” BPA exposure on telomerase activity and DNA repair capacity in activated human CD8 + T lymphocytes. (**A**) CD4 + and CD8 + T cell subsets were stimulated with anti-CD3/CD28 and exposed to BPA or solvent control (SC = DMSO 0.01%) for 24 h. Telomerase activity was analysed using the TRAP-ELISA assay. (**B**, **C**) Isolated CD8 + T cells were stimulated with anti-CD3/28 and treated with BPA or SC for 24 h. After that, the cells were washed with PBS before challenging with 5 µM H_2_O_2_ or 2.5% PBS (SC) for 10 min at 37 °C. 3 µM APC was used to block DNA damage repair in the cells. The % tail DNA was analysed as parameter for DNA damage and is given in (**B**), the calculated DNA repair capacity is given in (**C**). (**D**) Gene expression analysis of human DNA repair genes using a real—time PCR array. The scatter blot compares expression of 84 DNA repair pathway genes between the solvent control (0.01% DMSO) and 0.3 nM BPA treated cells. The solid diagonal line represents no change in expression. Any data point above the upper line represents genes that are upregulated > twofold. Any data point below the lower line represents genes that are downregulated > twofold. Bars are mean values; results were presented as means + SD. Significance of difference was calculated relative to the respective control, *p < 0.05; **p < 0.01.

Telomerase plays a critical role in the regulation of immune cell function and to further discern our previously reported findings on telomerase activity inhibition by BPA in CD3/CD28 stimulated PBMC^7^, we here first investigated the effect of BPA on telomerase in CD4 + and CD8 + T cell subpopulations. From Fig. 1A it can be seen that 24 h treatment of cells with BPA resulted in a non-monotonic telomerase enzyme inhibition in CD8 + T-cells, but not CD4 + T-cells of male donors. The maximum inhibition (30% as compared to solvent control) was seen at 0.3 nM BPA. At higher concentrations, the effect subsequently weakened. Based on these results, CD8 + T cells were used for subsequent investigations.

We next investigated the impact of BPA on repair capacity of CD3/CD28-activated CD8 + T-cells upon oxidative stress exposure. Similar to our observations on telomerase activity, DNA repair capacity was impaired at 24 h treatment with BPA as compared to the solvent control; the effect maximized at 0.3 nM (45% inhibition). The results of the comet assay, given as % Tail DNA, are depicted in Fig. 1B; the calculated %DRC is given in Fig. 1C. We next performed a DNA repair gene pathway analysis by using a human DNA repair RT^2^ Profiler PCR array. For this, CD8 + T cells were stimulated with CD3/CD28 together with 0.3 nM BPA for 24 h. However, no regulation on the expression of 84 repair genes upon BPA exposure could be observed (Fig. 1D). Also, no relevant modulation of intracellular as well as cell-surface thiol expression by BPA exposure could be detected in the samples (Supplement Fig. S1A and S1B).

### Long-term “low-dose” BPA exposure inhibited proliferation, telomere length, mtDNA copy number and hTERT protein expression in CD8 + T cells

**Figure 2:**
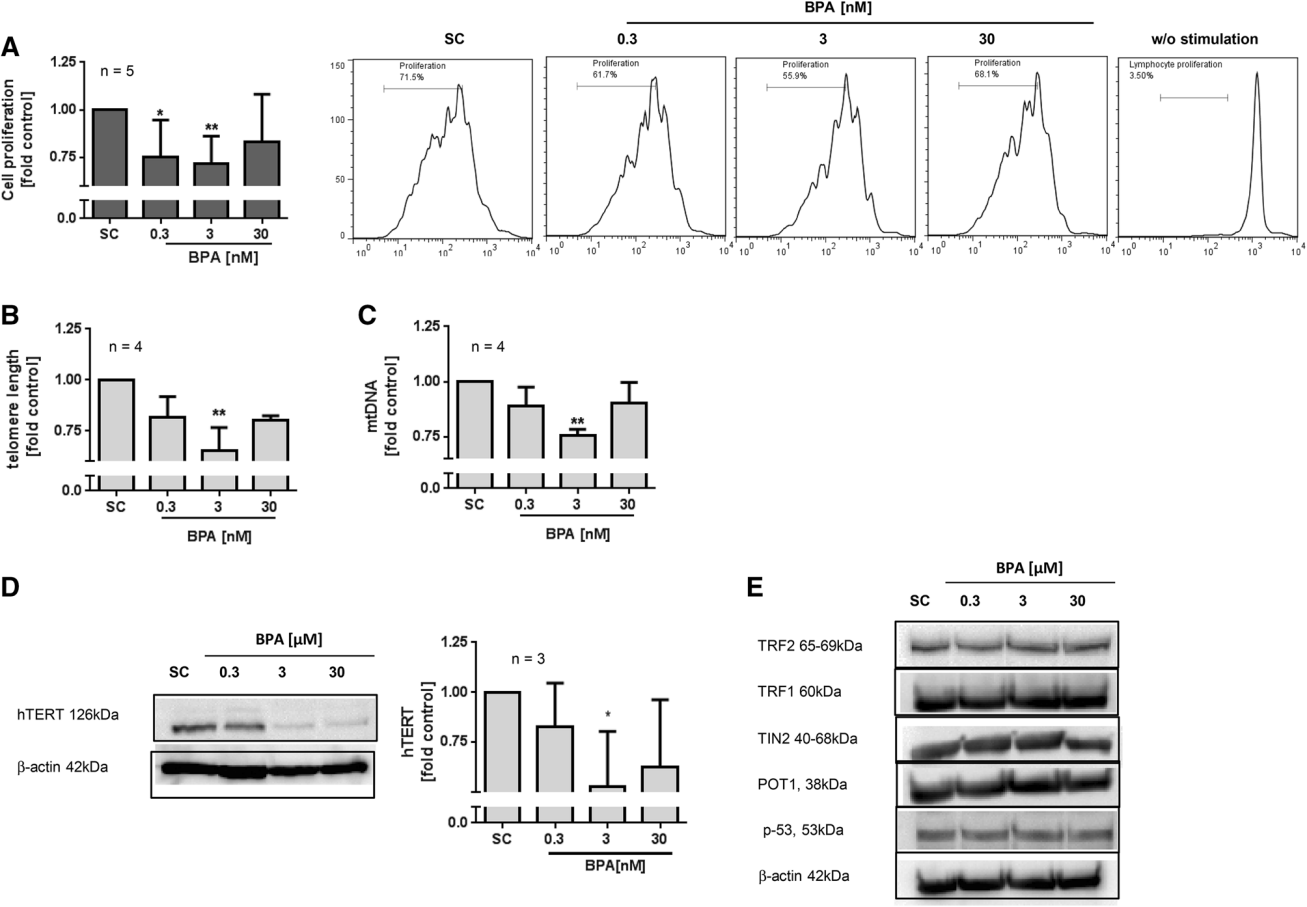
Effect of long-term BPA exposure on cell proliferation, telomere length and mtDNA copy number. Long-term culture of CD8 + T was done using T cell activation/expansion kit and IL-2. The cells were continuously exposed to BPA or solvent control (SC = 0.01% DMSO). (**A**) Cell proliferation was quantified using CFSE staining after 35d of culture. Cell proliferation was calculated relative to SC. Mean values + SD (left) and representative histograms are given (right). (**B–E**) Long-term exposed CD8 + cells were harvested after 49d of culture, washed twice and cell pellets were used for the analysis of (**B**) telomere length (**C**) mtDNA copy number analysis and (**D**-**E**) protein expression analysis. The picture depicts representative immunoblots of hTERT, TRF2, TRF1, TIN, POT1, p-53. β-actin was used for loading control. The graph (D, right) depicts the quantification of hTERT protein expression in BPA treated cells relatively to SC using by pixel densitometry. Bars are means + SD, *p < 0.05; **p < 0.01.

In order to investigate the long-term impact of BPA on cytotoxic CD8 + T lymphocytes, a long-term cell culture system was applied according to manufacturer’s instructions and as reported by Petersen et al.^30^. For this, CD8 + T cells, continuously exposed to BPA or solvent control, were treated with IL-2 together with anti-CD2/3/28 beads and re-stimulated every two weeks. Depending on the cell expansion period (every 3 or 7 days), the medium was replaced with fresh complete RPMI-1640 medium containing 20U/mL IL-2 and the test compounds. Proliferation of CD8 T-cells was then assessed on day 35 using CFSE staining. Long-term treatment with BPA significantly reduced cell proliferation at < 30 nM (Fig. 2A). The maximum inhibition was seen at 3 nM, with a reduction of 25% in comparison to the solvent control (raw data are provided in Supplement Fig. S2A). Whether long-term treatment of CD8 + T cells with BPA could indeed impair T cell response upon antigen stimulation was further investigated by studying telomere length and mtDNA copy number on day 49 of the long-term culture. Telomere length and mtDNA copy number are two widely used potential markers of biological age. Findings suggest a correlation of these two parameters in cellular aging and other age-related disorders such as cancer^31,32^. As demonstrated in Fig. 2B and Fig. 2C, BPA exposure shortened telomere length of T cells and also inhibited the number of mtDNA copies in the cells, at 3 nM BPA by 35% and 25%, respectively. At a tenfold higher or lower concentration, this effect was marginally evident for both parameters. Subsequently, the expression of the catalytic component of the telomerase complex (hTERT) and the telomere-associated proteins TRF1/2, TIN2, POT-1, and TPP1 as well as the p-53 proto-oncogene were analysed. From Fig. 2D it can be seen that among these proteins, only hTERT expression was noticeably suppressed by 45% at 3 nM. Corresponding to the observations on telomere length, BPA at 0.3 or 30 nM somewhat inhibited hTERT but the results were inconsistent. None of the other investigated proteins including TRF2, TRF1, TIN2, POT1 and p-53 (Fig. 2E) and TPP1 (data not shown) were affected by continuous BPA exposure at any tested concentrations as analysed by western blot.

### Long-term “low-dose” BPA exposure inhibited intracellular expression and secretion of IFN-γ in CD8 + T-cells

**Figure 3:**
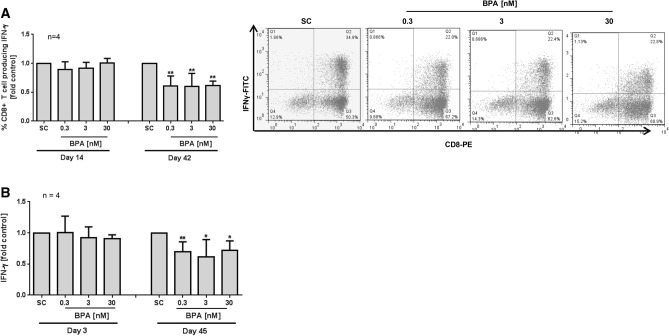
Long-term impact of BPA on cytokine levels of CD8 + T cells. Long-term culture of CD8 + T was done using anti-CD2/3/28 beads and IL-2. The cells were continuously exposed to BPA or solvent control (SC = 0.01% DMSO). (**A**) On day 14 or 42 of culture, CD8 + cells were stimulated with PMA/ionomycin and Brefeldin A for 4 h. Then, intracellular IFN-γ expression was analysed using anti-CD8-PE coupled with anti-IFN-γ FITC with a FACSCalibur. Representative scattergrams of intracellular IFN-γ expression at 42d from one experiment are shown. (**B**) Supernatants, harvested on day 3 or 45 of long-term culture were used for analysis of IFN-γ release. To correct for inter-individual differences, each value of BPA treated cells was normalized to its respective SC sample. Raw data are given in Supplement Fig. 2B, C. Bars are means + SD. Significance of difference was calculated relative to the respective control, *p < 0.05; **p < 0.01.

Cytokines play a crucial role in haematopoiesis as well as modulation of immune responses. Cytokine dysregulation is considered as indicator of “unsuccessful” ageing in the elderly and suggested to contribute to pathogenesis including cancer^33^. Thus, we investigated whether the decline of cytotoxic lymphocyte proliferation, telomere length and mtDNA copy numbers concurs with cell function impairment by quantification of anti-viral Interferon-γ (IFN-γ) expression and release in BPA exposed long-term CD8 + T-cell cultures. While no change could be seen on day 14 of BPA exposure at any tested concentrations, a strong inhibitory effect of intracellular IFN-γ expression was evident on day 42 of BPA exposure, as compared to the solvent control (Fig. 3A and Supplement Fig. S2B). In addition, supernatants of the cell cultures were collected at the indicated time points and IFN-γ release was quantified using ELISA technique. In line with intracellular IFN-γ expression, secreted IFN-γ levels did not change upon short-term exposure, but on day 45, a reduction of about 30% was seen at 3 nM BPA as compared to the solvent control (Fig. 3B and Supplement Fig. S2C).

### The observed effects are not due to a phenotypic shift in CD8 + T cells

**Figure 4:**
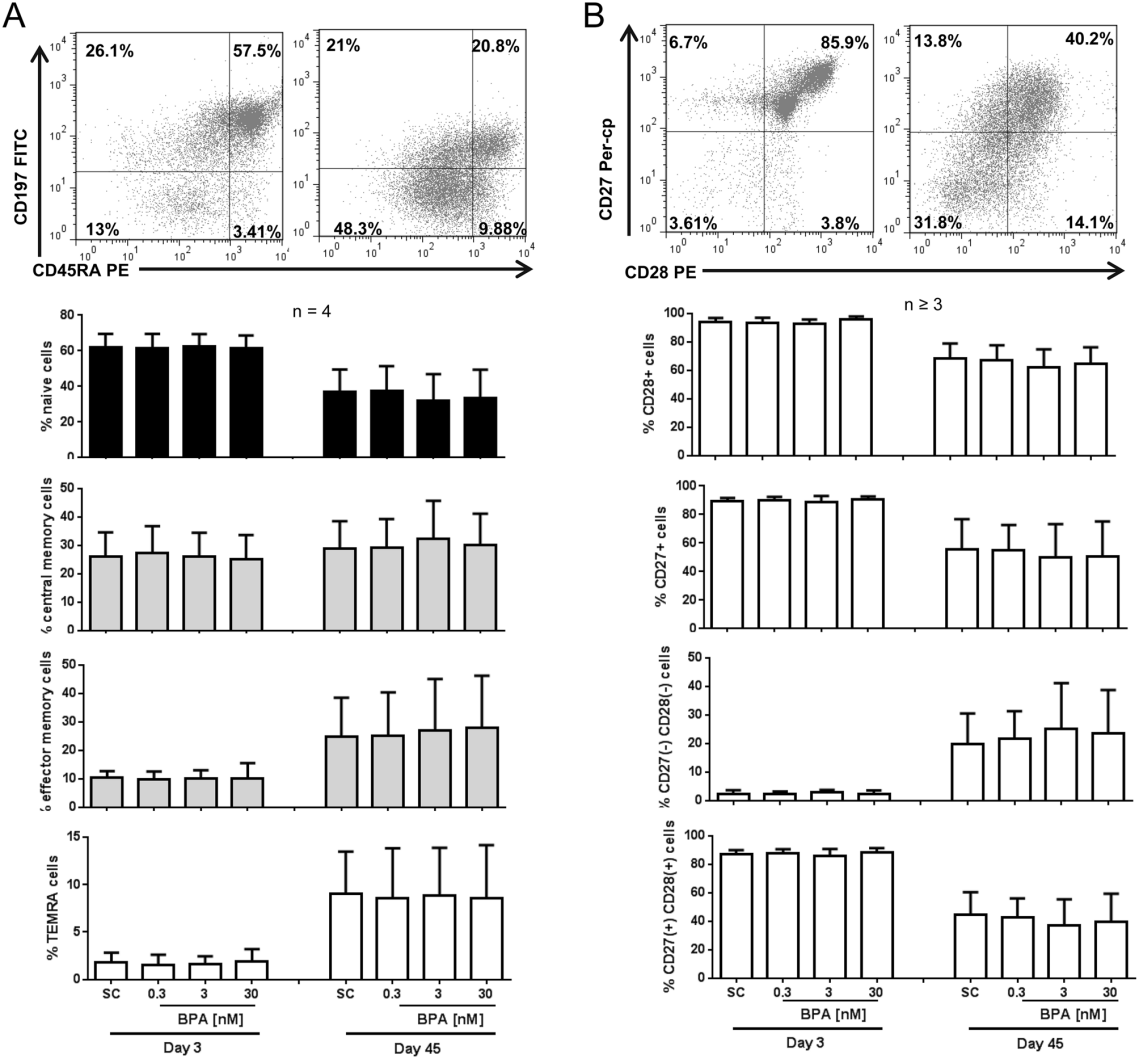
Phenotypic shift in long-term cultured CD8 + T cells. Long-term culture of CD8 + T cells was done using anti-CD2/3/28 beads and IL-2. The cells were continuously exposed to BPA or solvent control (SC = 0.01% DMSO). At day 3 and 45d, (**A**) the CD8 + T cell subpopulation was examined using the markers CD45RA + CD197 + for naive, CD45RA- CD197 + for central memory, CD45RA- CD197- for effector memory, CD45RA + CD197- for effector memory re-expressing CD45RA (TEMRA), (**B**) CD27/CD28 staining was used for determining the co-stimulator receptor expression. Representative scattergrams of different stains from one donor are shown. Bars are mean values; results were presented as means + SD of at least 3 independent experiments.

Since telomere length varies among naïve precursor, intermediate and end stage differentiated T cells^34^, we also assessed the phenotype of CD8 + T cells to determine whether the observed effects could be due to a phenotypic shift in the long-term culture by using the markers CD45RA + CD197 + for naive, CD45RA- CD197 + for central memory, CD45RA- CD197- for effector memory, CD45RA + CD197- for effector memory re-expressing CD45RA (TEMRA) and the co-stimulatory receptor markers CD27- CD28- for highly activated cells. Indeed, a phenotypic shift towards the accumulation of memory T cells is expected in long-term in vitro cell culture systems^35,36^. After 45 days in culture, the naïve CD8 + T cell population significantly decreased (in range of 40% reduction), while memory subsets increased with substantial elevation in the effector and TEMRA cells (Fig. 4A). Likewise, the CD28 + /CD27 + co-stimulatory receptor expression was reduced after repeated rounds of re-stimulation; while after 3 days most of the CD8 + T cells (> 80%) were able to activate and express CD28 + /CD27 + receptors (Fig. 4B), on day 45, a strong reduction of CD27 + CD28 + cells with an increase in highly differentiated CD28-CD27- cells (both by about 40%) was observed (Fig. 4B). However, interestingly, no difference was evident between the phenotype of CD8 + T cells of BPA and solvent treated samples.

## Discussion

Shortened leukocyte telomere length and telomerase activity dysfunction have been linked with increased mortality and age-related disorders including cancer^27,28,37^. Also, telomere shortening in CD4 + and CD8 + T cells has been suggested to be relevant in the decline of T cell function^34,38^. Thus, nowadays, shorted telomeres are considered as prognostic marker of accelerated ageing, premature mortality or even incidence of cancer^39,40^. Interestingly, a cross-sectional study published in 2019 reported on a potential association of higher urinary BPA levels with shorter peripheral leukocyte telomere length in adult Lebanese females. This observation still persisted after correction for confounders including smoking, drinking, or a diagnosis of cancer^41^. Of course, this cross-sectional study has its drawbacks, one of it being a relatively small sample size of 501 volunteers. There are further limitations due to the single urinary sample collection which does not consider potential BPA level variability over time and of course also the inability to assess for the longitudinal association of BPA with telomere length due to the study design^41^. Study replication using better designs and further investigations are here needed before any conclusion can be drawn.

In our in vitro study, we now found reduced telomere length and telomerase activity in CD8 + T cells from healthy donors upon long-term “low-dose” BPA exposure. A rapid, non-classical ER/GPR30-ERK down-stream signal transduction has already been proposed by us as the underlying mechanism of telomerase activity inhibition in stimulated PBMC^7^. This was then demonstrated by successful abolishment of BPA mediated telomerase inhibition using specific antagonists for ERα and β, GPR30, and also for the MAPK kinase ERK1/2. One mechanism of telomerase activity regulation is control of hTERT transcription, but we could not confirm that BPA affected hTERT mRNA expression in PBMC in our previous study^7^. So we rather suggest that activity regulation occurs via different mechanisms, i. e. at the level of telomerase holoenzyme assembly^42^, via hTERT translocation or hTERT mRNA splicing.

Cytotoxic CD8 + T lymphocytes are one of the major cell populations that regulate the immune response to pathogens as well as tumour cells^43^. The CD8 + T cell response mainly depends on the capability of clonal lymphocyte expansion. To a certain extent, telomerase can here compensate for telomere loss in T cell proliferation and thus is vital for regulating T cell replicative lifespan^38,44,45^. It also enhances immune cell function whereby impaired telomerase might indicate early deterioration of the immune system^46^. The catalytic component of telomerase (hTERT) plays a decisive role for enzyme activity; higher levels of hTERT in activated T cells have been correlated to enhanced expression of telomerase activity and increased proliferative capacity of T cells in vitro^45^. Besides telomerase, shelterin, i. e. a complex formed by six telomere-specific proteins (TRF1/2, POT1, TIN2, RAP1 or TPP1) has an important function in telomere heterochromatin maintenance by protecting chromosome ends. Without the protective activity of shelterin, telomeres are no longer hidden from the DNA damage surveillance and chromosome ends are inappropriately processed by DNA repair pathways^47^. Based on our findings, we suggest that depletion of the hTERT protein but not shelterin complex proteins may account for the observed CD8 + T cell telomere shortening by BPA.

Beyond the canonical function of telomerase, there is limited evidence that TERT can bind to mtDNA through the coding regions of the NADH:ubiquinone oxidoreductase subunits and it has been suggested that the reverse transcriptase activity may be relevant for protecting mtDNA^48^. This concurs with our observation of reduced mtDNA copy numbers in long-term cultured CD8 + cells. In human studies, a positive correlation between leukocyte mtDNA copy number and telomere length has been reported^49^. Thus, emerging studies extend the association of leukocyte telomere length, telomerase activity and mtDNA copy number to immune senescence and cancer risk^31,32,50^.

Release as well as intracellular IFN-γ production was suppressed in BPA treated CD8 + T cells in the present study. So far, several studies on mice demonstrated that an age-associated decline in CD8 + IFN-γ levels is correlated with decreased cytotoxic function, cell senescence, and increased susceptibility to viral infection as well as cancer, whereas enhancement of IFN-γ production could elevate the anti-tumour response^51–53^. The IFN-γ promoter also contains estrogen response elements (EREs), hormonal factors could thus be direct regulators of IFN-γ gene expression^54^. Animal studies suggested BPA could also modulate the immune response through IFN-γ, and its long lasting effects could be due to ERE promoter regulation^55^. This concurs with a study from Roy et al.^56^. Here, maternal mice exposure to BPA resulted in a decreased level of IFN- γ mRNA expression in offsprings with influenza virus infection. In the present study, long-term BPA treatment resulted in IFN-γ down-regulation along with other parameters. However, this could not be observed upon short-term BPA exposure. Thus, IFN-γ suppression might be rather a consequence of proliferation and telomere length attrition.

It has been shown that the T cell repertoire changes with advanced ageing or under long-term culture conditions in vitro^35,36,57^. As expected, the CD8 + T cell long-term cultures showed a phenotypic shift towards higher differentiated lymphocytes together with lower expression of CD28 + /CD27 + co-stimulatory receptors. However, there was no difference seen in T cell frequency between control and BPA treated cultures. So, the observed effects in BPA-treated cultures are rather not a consequence of the long-term culture conditions.

BPA has been shown in vitro and in animal models to exhibit a non-monotonic dose response (NMDR) which has been described for regulation of protein kinases, transcriptional activity, cell proliferation, metabolism, carcinogenesis and metastasis^12,58^, but data are inconsistent so far. Possible explanations for the NMDR include various physiological mechanisms with complexicity, e.g. involvement of distict receptor expression levels, dose-dependent receptor affinities, or regulatory feedback loops^12,59,60^. Furthermore, the concept of NMDR relationship remains elusive in the human population due to limited evidence.

In conclusion, the results are in accordance to our previous data on PBMC and provide more insight into the potential adverse effects of “low-dose” BPA to human immune cells. The results on telomerase activity indicate to different levels of sensitivity of T cell subpopulations to BPA. For most analysed parameters, a clear non-monotonic concentration response curve could be observed. This is in line with many other in vitro and in vivo studies on BPA^61^. There is of course a limit to the information that can be obtained by in vitro studies and whether these observations are transferable to in vivo is so far inconclusive. Together with the negative association found between BPA exposure and peripheral telomere length as reported in a cross-sectional study^41^ these results might merit some special attention to further investigate BPA and the risk of immune disorders in terms of adaptive immune response dysfunction and early onset of cancer in man.

## Material and methods

### Materials

DMSO (purity > 99%), acrylamide (30%, Mix 37, 5:1) and bovine serum albumin (BSA) fraction V were from Applichem GmbH (Darmstadt, Germany). β-mercaptoethanol was purchased from Fluka (Buchs, Switzerland). L-glutamine and phosphate buffered saline (PBS, without Ca and Mg), penicillin/streptomycin (P/S) solution and RPMI-1640 were from Life Technologies Invitrogen (Darmstadt, Germany). Fetal calf serum (FCS), charcoaled stripped, was from Biochrom (Berlin, Germany) and nuclease free water from Qiagen (Hilden, Germany). Phorbol 12-myristate 13-acetate (PMA), ionomycin (calcium salt) and brefeldin A were obtained from Cayman (Hamburg, Germany). Triton X-100 (molecular biology grade), tris (hydroxymethyl) aminomethane (Tris, Pufferan, p.a., 99.9%) and sodium hydroxide (≥ 99%, p.a.) were purchased from Carl Roth (Karlsruhe, Germany). Bisphenol A (> 99%), Tween-20, protein standard BSA, ammoniumpersulfate, hydrogen peroxide (H_2_O_2_, 30% w/w) solution in water with stabilizer and ethidium bromide (BioReagent, for molecular biology, 10 mg/ml in H_2_O) were purchased from Sigma-Aldrich Chemie GmbH (Taufkirchen, Germany). Low melting point agarose (LMPA), normal melting point agarose (NMPA) and ethyl diamine tetra acetic disodium salt (EDTA, 99%, p.a.) were obtained from Serva GmbH (Heidelberg, Germany). ( +)- aphidicolin (APC, > 98%), isopropyl alcohol (≥ 99.7%) and hydrochloric acid (37%) were purchased from VWR Chemicals (Bruchsal, Germany), absolute ethanol (≥ 99.8%, p.a.) and acetone (p.a. ≥ 99.8%, p.a) were from Fisher Scientific (Schwerte, Germany). Anti-human CD3 and CD28 functional grade purified antibodies were from eBioscience Affymetrix (Frankfurt, Germany). Recombinant human Interleukine-2 (IL-2) and human T cell activation/expansion kit were purchased from Miltenyi Biotec GmbH (Bergisch Gladbach, Germany). Amersham ECL Select and Hybond ECL Nitrocellulose Membrane were obtained from GE Healthcare Biosciences AB (Uppsala, Sweden), Quick Start Bradford 1 × Dye Reagent was from BioRad Laboratories GmbH (Munich, Germany) and PageRuler Plus Prestained Protein ladder from Thermo Fisher Scientific (Waltham, Massachusetts, USA). The following primary antibodies were used for immunoblotting: anti-telomerase reverse transcriptase antibody (h-TERT, C-terminal, ab 183,105,1:500) was purchased from Abcam (Cambridge, UK); anti-TRF-2 (9F10,1:1,000) and POT1 (M1-P1H5,1:1,000) were purchased from Santa Cruz (Heidelberg, Germany); anti-TIN2 (59B388.1, 1:1,000) was purchased from Bio-Techne GmbH (Wiesbaden-Nordenstadt, Germany) , p-53 (9,282,1:1,000) was from Cell Signalling (Danvers, Massachusetts, USA), and anti-beta-actin (clone AC-74,1:10,000) from Sigma-Aldrich Chemie GmbH (Taufkirchen, Germany). The horseradish peroxidase (HRP)-labelled secondary antibodies anti-mouse and anti-rabbit were purchased from Cell Signalling (Danvers, Massachusetts, USA). The following primary human antibodies labelled with fluorophore were used for flow cytometry: CD8‐PE/APC (clone BW135/80), CD4‐PE/APC (clone REA623), CD3-FITC/PE/APC (clone REA 613), CD27-PerCP (clone M-T271), CD28-FITC/PE (clone REA 612), CD45RA-PE (clone T6D11), CD197-FITC (clone REA 108), IFN-γ-FITC (clone REA600) (Miltenyi Biotec, Bergisch Gladbach, Germany). Carboxyfluorescein diacetate succinimidyl ester (CFSE) was purchased from eBioscience (Frankfurt, Germany). Forward and reverse primers of telomere and 36B4 gene were obtained from Biomers (Ulm, Germany).

### Isolation of human CD4 + and CD8 + T cells from volunteers’ blood or from buffy coat

Blood samples were drawn in the morning from 8 to 11 AM (n = 25), in Li-Heparin vacutainers from healthy volunteers at the University of Freiburg—Medical Center after written informed consent. At the time of blood sampling, volunteers (male donors, aged between 22 to 42 years) had a normal BMI, were healthy and did not take any medication. CD4 + or CD8 + T lymphocytes were isolated from blood using the EasySep Direct Human CD4 + /CD8 + T Cell Isolation Kit (StemCell Technologies, Cologne, Germany) according to the manufacturer’s instructions.

For long-term culture, CD8 + lymphocytes were isolated from buffy coats obtained from the Blood Transfusion Centre (Freiburg University Medical Center, Germany). Briefly, PBMC isolation was done by centrifugation on a LymphoPrep gradient (density: 1.077 g/cm^3^, 20 min, 500xg), cells were then washed twice with PBS and CD8 + T cell subsets were further isolated using an EasySep Human CD8 + T Cell Isolation Kit (StemCell Technologies, Cologne, Germany). Cell viability and concentration was determined using the trypan blue exclusion test. CD8 + T cells (1 × 10^6^ cells/mL) were cultured in phenol red free RPMI-1640 medium supplemented with 10% heat-inactivated charcoal-treated FCS, 2 mM L-glutamine, 100 U/mL penicillin/streptomycin, at 37 °C, in a humidified incubator with a 5% CO_2_/95% air atmosphere.

### Long-term culture of CD8 + T lymphocytes

Long-term CD8 + T cell culture was applied according to the manufacturer’s instructions and as described by Petersen et al.^30^. Briefly, CD8 + T cells were maintained at a concentration of 1 × 10^6^ cells/mL in culture media supplemented with 20U/mL IL-2 and the respective test compounds (BPA or 0.01% DMSO). Cells were split every three to seven days with addition of 20U/mL IL-2 and test compounds; each 14 days, cells were re-stimulated using the human T Cell Activation/Expansion Kit (Miltenyi Biotec, Bergisch Gladbach, Germany). T cells were cultured for a total of 49 days; during this time, aliquots were taken on a regular basis from each cell culture for determination of effect parameters. At the end of the long-term culture cell pellets were stored at -80 °C until further processing.

### Analysis of cell proliferation using CFSE staining

Cell proliferation was analysed as described previously^7^. In short, cells (5 × 10^6^ cells/ml) were washed once with PBS, resuspended in PBS supplemented with 5% heat inactivated, charcoaled treated FCS and then incubated for 10 min at room temperature (RT) with 5 µM CFSE. Culture medium was added and cells incubated for 5 min on ice before washing with culture medium for two times. Cell proliferation was analysed on day 35 of culture using a FACSCalibur (BD Biosciences, Heidelberg, Germany).

### Telomerase activity measurement by TRAP-ELISA assay

Telomerase activity was detected using the *TeloTAGGG* Telomerase PCR ELISA Kit, from Roche (Mannheim, Germany) as described before^62^. In brief, 0.5 × 10^6^ cells were lysed according to the manufacturer’s protocol. An equal amount of protein as determined by the Bradford method^63^ was added to the reaction mixture to a final volume of 50 µl. The reaction was performed in an Eppendorf Mastercycler Nexus Thermal Cycler (Thermo Fisher Scientific GmbH, Dreieich, Germany) following these steps: 25 °C for 20 min, then denatured at 94 °C for 5 min, 30 cycles (94 °C for 30 s, 50 °C for 30 s and 72 °C for 90 s), followed by elongation at 72 °C, 10 min. A heat-treated sample (95 °C for 5 min) was used as negative control. Subsequently, 5 µl of the PCR product were denatured and hybridised with adeoxigenin-labelled telomeric repeat probe. Absorbance was measured at 450 nm (reference 690 nm) using a multiplate reader from Tecan (Tecan Group Ltd, Crailsheim, Germany).

### RNA isolation

Total RNA was isolated from 4 × 10^6^ CD8 + T cells by using the RNeasy mini Isolation kit from Qiagen (Hilden, Germany) followed by a purification step using the RNase-free DNase kit from Qiagen (Hilden, Germany) according to the manufacturer’s instructions^42^. RNA quality and quantity were assessed using a NanoDrop ND-1000 spectrophotometer (Thermo Scientific, Freiburg, Germany).

### Human DNA repair RT-PCR array

1 µg RNA was taken for cDNA synthesis using the RT^2^ PCR array First Strand Synthesis Kit (Qiagen, Hilden, Germany). The qPCR of RT^2^ Profiler PCR Array Human DNA Repair (cat no: PAHS-042Z from Qiagen, Hilden, Germany) was performed using a CFX96 Touch Real-Time PCR Detection System (Bio-Rad, Müchen, Germany). The PCR was carried out at 95 °C for 15 min, followed by 40 PCR cycles (95 °C for 10 s, 60 °C for 15 s, rampe rate 1 °C/s) and a final extension for 5 min at 72 °C, followed by a standard melting curve analysis. The web-based automated RT^2^ Profiler PCR Array Data Analysis from the manufacturer was used to analyse the data (https://geneglobe.qiagen.com/us/analyze/).

### DNA isolation

Genomic DNA was isolated from 1.5 × 10^6^ CD8 + T cells using the FlexiGene DNA Kit from Qiagen (Hilden, Germany) according the manufacturer's instructions. DNA purity and quantity were determined using a NanoDrop ND-1000 spectrophotometer (Thermo Fisher Scientific GmbH, Dreieich, Germany).

### Telomere length quantification

Full telomere length was quantified based on the method of O'Callaghan and Fenech^64^ using the 36B4 gene as reference. Each sample contained 20 ng of purified DNA, 2 × Maxima SYBR Green qPCR master mix (Thermo Fisher Scientific GmbH, Dreieich, Germany), forward and reverse primers for the telomere or 36B4 gene. Each sample was run in triplicate using a CFX96 Touch Real-Time PCR detection System (Bio-Rad, Munich, Germany). The cycling conditions were set at 95 °C for 10 min, followed by 40 PCR cycles (95 °C for 15 s, 60 °C for 1 min), and subsequent standard melting curve analysis. Data were analysed using the comparative ΔΔC_T_ method calculating the difference between the threshold cycle (C_T_) values of the target and reference gene of each sample and then comparing the resulting of the ΔC_T_ values between the different samples.

### Mitochondrial DNA copy number quantification

Relative quantification of human mitochondrial DNA copy number was done using the Relative Human Mitochondrial DNA copy number quantification qPCR Assay kit (Provitro AG, Berlin, Germany) according to the manufacturer’s instructions. Briefly, 5 ng genomic DNA were mixed with 2 × Maxima SYBR Green qPCR master mix ( Thermo Fisher Scientific GmbH, Dreieich, Germany) and mtDNA primer or the single copy reference (SCR) primer set. Quantitative PCR of 20 µl total reaction volume was performed at 95 °C for 10 min, followed by 40 PCR cycles (95 °C for 29 s, 52 °C for 20 s, 72 °C for 45 s) and subsequent melting curve analysis. Data analysis was done as describe above.

### Flow cytometry

Purity analysis of freshly isolated CD8 + T cells (2 × 10^5^) was done using an anti-CD8-APC mAb. Only T cell populations with a purity > 90%, as determined by flow cytometry, were used for the experiments. For identifying co-stimulatory receptor expression, CD8 + T cells (2 × 10^5^) were stained with anti-CD8-APC mAb coupled with anti-CD28-PE and anti-CD27-PerCP mAbs for 30 min at 4 °C. Staining for naive and memory cells was performed using anti-CD8-APC and anti-CD45RA-PE together with anti-CD197-FITC mAbs. Cells were washed twice with PBS buffer containing 0.5% charcoaled treated FCS and 2 mM EDTA prior to analysis by flow cytometry.

For analysis of intracellular IFN-γ, cells (2 × 10^5^) were stimulated with 50 ng/ml PMA, 1 µg/ml ionomycin and 10 µg/ml brefeldin A for 4 h, then washed twice with PBS buffer containing 0.5% charcoaled treated FCS and stained with anti-CD8-PE mAb for 30 min at 4 °C. Cells were then fixed and permeabilized using the BD Cytofix/Cytoperm Fixation/Permeabilization Kit (BD Biosciences, Heidelberg, Germany) according to the manufacturer's protocol before staining with anti-IFN-γ-FITC mAb. Afterwards, the cells were analysed by flow cytometry. All experiments were analysed using a FACSCalibur.

### Protein analysis by immunoblotting

Protein expression was analysed by immunoblotting as described before^42^. Briefly, 40 µg of protein, as determined by the Bradford method^63^ were separated by SDS-PAGE polyacrylamide gels and then transferred to a nitrocellulose membrane by wet blotting (0.7 mA/cm^2^, 90 min.). The membranes were blocked with 5% low fat milk in TBS/Tween 0.1% and probed with primary antibodies overnight at 4 °C or 1 h at RT, followed by incubation with secondary antibodies conjugated with horseradish peroxidase for 1 h at RT. The membranes were developed with an enhanced chemiluninescence substrate. Subsequently, the protein signals were imaged by the gel documentation system Molecular Imager ChemiDoc XRS sytem (Bio-Rad, Munich, Germany) and normalised to the corresponding β-actin.

### Quantification of DNA damage and repair capacity

DNA damage and repair capacity of CD8 + T cells was performed as described by Odongo et al.^65^. Briefly, CD8 + T cells (5 × 10^4^) were exposed to the test compounds for 24 h. After that, cells were resuspended and 50 µl of cell suspension transferred to a 96-well v-bottom plate (Sarstedt, Nümbrecht, Germany), centrifuged (5 min, 500xg), and the supernatant discarded. Afterwards, cells were resuspended in 100 µl RPMI-1640 medium supplemented with 2 mM L-glutamine, 100 U/mL penicillin/streptomycin with or without the addition of 5 µM H_2_O_2_ and incubated for 10 min at 37 °C. Then, 150 µl of 10% cold DMSO diluted in PBS were added to the cells to stop the H_2_O_2_ reaction. The plates were centrifuged (500 × g, 5 min, 4C°), and the supernatant was discarded. Cells were resuspended in 50 µl medium with or without the addition of 3 µM APC and incubated for 1 h before processing for comet assay as described in Odongo et al.^65^. Cells were analysed using a Leica fluorescence microscope (Leica DMLS, excitation filter: BP 546/10 nm, barrier filter: 590 nm) connected to an image analysis system (Comet 5.5, Optilas GmbH, Müchen, Germany) after staining with 75 µl ethidium bromide solution (10 µg/ml in ddH_2_O). For each sample, 100 cells were analysed. The percent tail DNA was used as indicator of DNA damage. Hedgehog comets were excluded from the analysis. The percentage DNA repair capacity (% DRC) was calculated as

$$ \% {\text{ DRC }} = \frac{{(\% \;{\text{Tail}}\;{\text{DNA}}\;{\text{H}}_{2} {\text{O}}_{2} + {\text{APC)}} - (\% \;{\text{Tail}}\;{\text{DNA}}\;{\text{H}}_{2} {\text{O}}_{2} )}}{{(\% \;{\text{Tail}}\;{\text{DNA}}\;{\text{H}}_{2} {\text{O}}_{2} + {\text{APC)}} - (\% \;{\text{Tail}}\;{\text{DNA}}\;{\text{APC)}}}} \times 100\% $$

### Quantification of cytokine release by ELISA Assay

Cell supernatants were collected at different time points. The human IFN-γ ELISA Ready-Set-Go kit supplied from eBioscience (Frankfurt, Germany) was used to quantifiy IFN-γ release according to the manufacturer's instructions.

### Statistics

Results were analysed using the GraphPad Prism 6.0 software (La Jolla, California, USA). Data are presented as means + SD. Statistical significance was determined by the ordinary one-way ANOVA followed by Bonferroni correction. *P* values < 0.05 (*) were considered statistically significant and < 0.01 (**) were considered highly statistically significant.

### Ethical approval

The study was approved by the Ethics Committee of the University of Freiburg and carried out according to their guidelines and regulations (No: 428/16 and 384/12).

## Supplementary information

Supplementary file1

Supplementary file2

## Data Availability

All data generated or analyzed during this study are included in this published article (and its Supplementary Information files).
