# Supplementary figures and images for: Long-term exposure to “low-dose” bisphenol A decreases mitochondrial DNA copy number, and accelerates telomere shortening in human CD8 + T cells
Source: Sci Rep. 2020 Sep 25;10:15786. doi: 10.1038/s41598-020-72546-x (PMC7519100; doi:10.1038/s41598-020-72546-x)

Figure S1

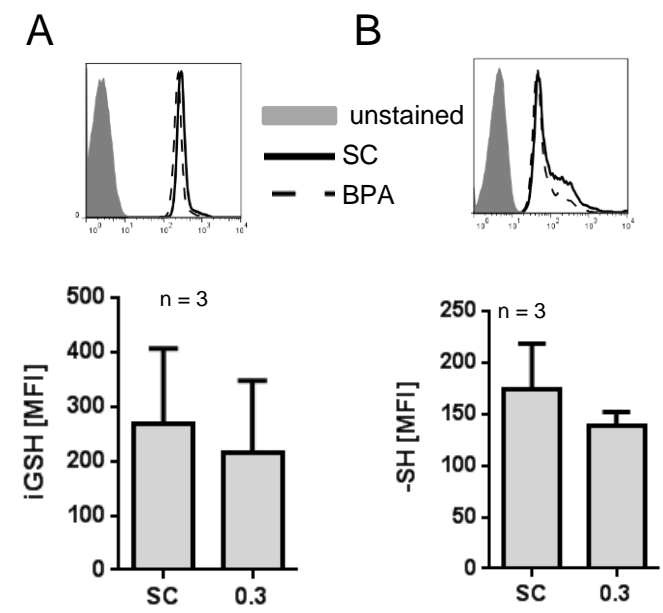

Figure S2

A

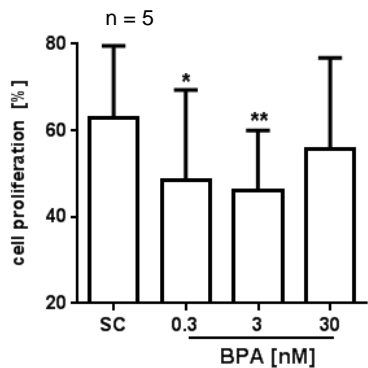

B

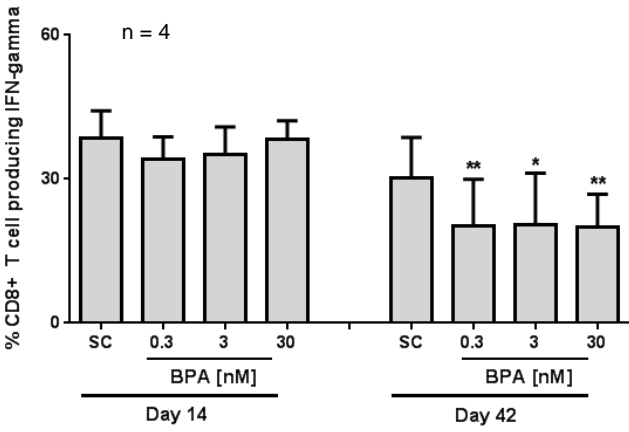

C

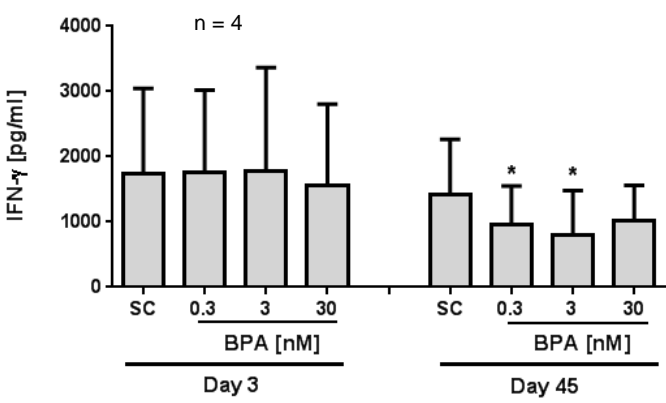

Figure S3

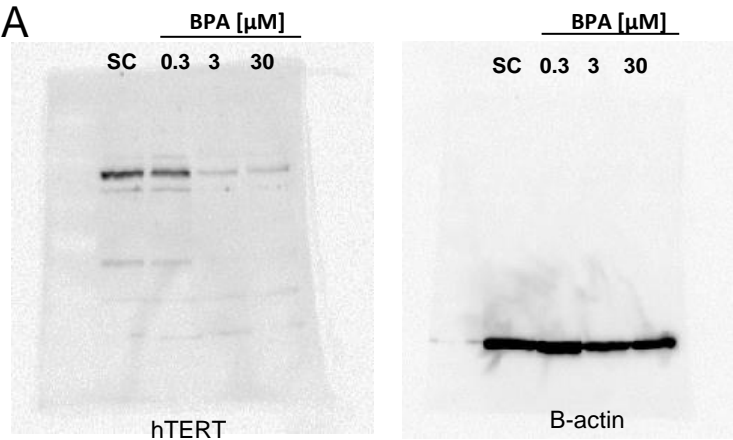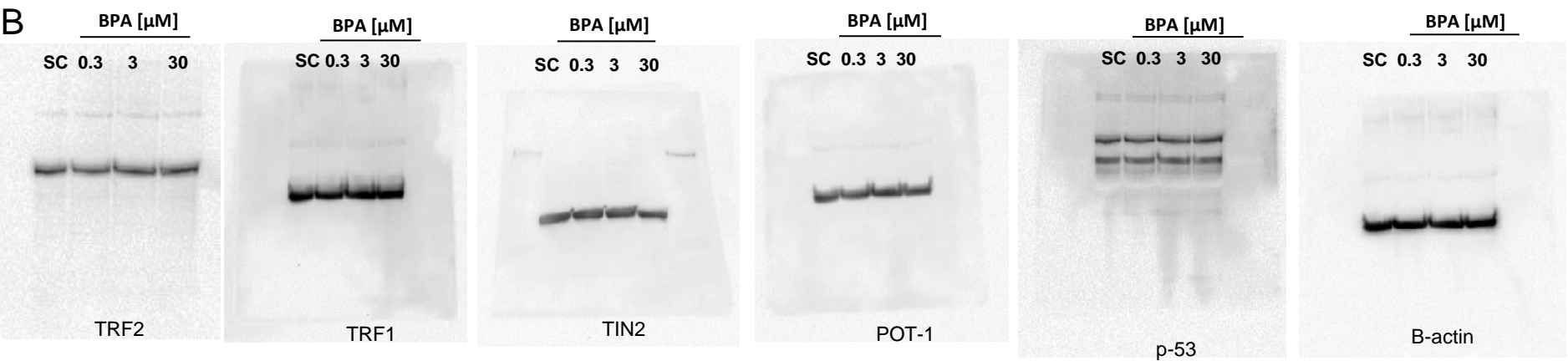

Supplement: Supplementary file 1 — Supplementary file1 [file 41598_2020_72546_MOESM1_ESM.pdf]
