# Supplementary material for: Long-term exposure to “low-dose” bisphenol A decreases mitochondrial DNA copy number, and accelerates telomere shortening in human CD8 + T cells
Source: Sci Rep. 2020 Sep 25;10:15786. doi: 10.1038/s41598-020-72546-x (PMC7519100; doi:10.1038/s41598-020-72546-x)
Supplement: Supplementary file 2 — Supplementary file2 [file 41598_2020_72546_MOESM2_ESM.pdf]

Long-term exposure to “low-dose” bisphenol A decreases mitochondrial DNA copy number, and accelerates telomere shortening in human CD8+ T cells

Hoai Thi Thu Tran[1, 2], Corinna Herz[1] and Evelyn Lamy[1]*

[1]Molecular Preventive Medicine, University Medical Center and Faculty of Medicine – University of Freiburg, 79106 Freiburg, Germany

[2]Pharmaceutical Bioinformatics, Institute of Pharmaceutical Sciences, Faculty of Chemistry and Pharmacy, Albert-Ludwigs-University, Freiburg, Germany

*corresponding author: Prof. Dr. Evelyn Lamy

evelyn.lamy@uniklinik-freiburg.de;

Tel.: +49(0)76127082150

Supplementary Materials and Methods

Materials

The following primary antibodies labelled with fluorophore or dyes were used for flow cytometry: green 5-chloromethylfluorescein diacetate (CMFDA) from Biomol GmbH (Hamburg, Germany), Alexa Fluor™ 488 C5 maleimide from Thermo Fisher Scientific (Freiburg im Breisgau, Germany) and IFN-γ-FITC (clone REA600) from Miltenyi Biotec (Bergisch Gladbach, Germany).

Intracellular glutathione and surface thiol expression

CD8+ T cells (2×10^5) were stained for intracellular and surface thiol expression using thiol reactive probes. After treatment with BPA or solvent control (SC=0.01% DMSO) for 24h, cells were incubated with 1µM CMFDA for 20min in an incubator 37°C, 5% CO_2 before analysis using flow cytometry. For cell-surface thiols, CD8+ T cells were stained with 5µM Alexa Fluor™ 488 C5 Maleimide for 2h at RT. Cells were then washed twice with PBS and analysed using flow cytometry. The median fluorescence intensity (MFI) of each sample was recorded using the FlowJo software (Ashland, Oregon, USA).

Supplementary Figure Legends

Supplementary Figure S1: Isolated CD8+ T cells were stimulated with anti-CD2/3/28 and treated with BPA/solvent (0.01%DMSO) for 24h. **A)** Intracellular glutathione and **B)** surface thiol expression were analysed using flow cytometry. A representative staining from one sample is shown. Bars are mean values; results were presented as means + SD.

Supplement Figure S2: Activated CD8+ T cells using anti-CD2/3/28 beads and IL-2 were constantly cultured with BPA or solvent. **A)** Cell proliferation was quantified by CFSE staining after 35d. **B)** Cells at 14d or 42d were then stimulated with PMA/ionomycin and brefeldin A for 4h. The percentage of CD8+ T cells with positive intracellular IFN-γ expression was evaluated using anti-CD8 PE coupled with anti-IFN-γ FITC in a FACSCalibur. **C)** Supernatants were collected from long-term BPA or solvent treated CD8+ T lymphocytes and prepared for analysis of IFN-γ release at the indicated time points. Bars are means + SD, significance of difference was calculated relative to the respective control *p<0.05; **p<0.01.

Supplement Figure S3: The pictures depict original immunoblots of (**A**) hTERT or (**B**) TRF2, TRF1, TIN2, POT1 and p-53. Long-term exposed CD8+ T cells were harvested after 49d of culture, washed twice and total lysate was subjected to immunoblotting. β-actin was used as loading control.
